# Chlorate of Potash in the Treatment of Erysipelatous Inflammations of the Mouth and Fauces; Vulgo, Black Tongue

**Published:** 1849-04

**Authors:** R. L. Scruggs

**Affiliations:** Germantown, Tenn.


*Chlorate of Potash in the Treatment of Erysipelatous Inflammations of the Mouth and Fauces; Vulgo, Black Tongue.* By R. L. SCRUGGS, of Germantown, Tenn.

When, several years ago, I settled in this village, with the view of practising my profession, I heard a great deal said abou a disease, then prevalent here, much dreaded both by people and physicians, called by the vulgar, "*Black Tongue.*"

It had, in many places, proved very fatal, and those who recovered from it, suffered intensely, partly from the effects of the disease, but chiefly from the dread of its malignancy.

In endeavouring to decide upon some plan of treatment that would seem to offer some better hope of success than that hitherto resorted to, I bethought me of the article chlorate of potash, as used by Watson and others, in the treatment of scarlatina maligna, and to my great delight found it to answer a most admirable purpose.

My first patient, an old lady aged about 55, presented the disease in its worst form. She had been attacked several days previously, with shivering, followed by fever, sore throat and swelling of the parotid and sub-maxillary salivary glands. The erysipelatous inflammation, commencing at the pharynx, had extended itself to all parts of the mouth, the nose, and probably the sinuses communicating with it, stuffing up the nostrils so completely, that the patient could only breathe through the mouth. Passing up the lachrymal ducts, it had involved the eyes, and out at the mouth and nose, involving the face, ears and part of the scalp. The external parts had been treated with scarifications, cauterizations with argt. nitr., and the application of tinct. iodine and solution of sulph. iron. This, with the disease, (the pus and sanies running out of the nose, eyes and the corners of the mouth,) had completely metamorphosed the "human face divine," rendering it more hideous than the worst case of confluent small pox. The tongue was protruding from the mouth, the upper surface of which was *black*, dry and chapped, and she was unable to articulate a word.

After using a tolerably strong solution of the chlorate, (a tea spoonful of the salt, to half a tea cup full of water,) for ten or fifteen minutes, thus cleansing the mouth of a quantity of offensive sloughy matter, she was enabled to speak indistinctly, but sufficiently well to let us know that it had afforded great relief. A tea spoonful of the chlorate was then put into a pint of water, to be used as a drink in the next 24 hours—a swallow or two at a time; with directions to cleanse the mouth each time before drinking, with the stronger solution. This, with the treatment previously instituted, caused a rapid convalescence, and the old lady was soon well.

Several of this family had the disease in quick succession, and it was carried to other families in the neighbourhood, by persons who visited them during their illness. Some of these cases were mild, others severe. In some the erysipelatous inflammation was confined to the skin, in others the mucous membrane of the mouth was the only part affected, and in all of these latter cases the chlorate evidently exercised a controlling influence from the beginning, or at any stage of the disease in which it was used.

How this agent acts locally, or upon the constitution, I shall not attempt to determine; its modus operandi, I must leave to others more competent to decide than me. My own experience of its efficacy in the treatment of this particular disease, is all that I have attempted in this short notice; and I venture to recommend it to my professional brethren, confidently believing that they will find it a most valuable adjuvant in the treatment of the much dreaded "Black Tongue." I have used it in the treatment of typhoid fevers; and, indeed, I am in the habit of employing it in almost all cases where the mouth and tongue are found to be dry, under the impression that it has power to relieve this unpleasant condition, but in what way I am not prepared to say, probably by promoting the secretions of the muciparous and salivary glands. Although I have been pleased with its action in these cases, I have never found its beneficial effects so marked as in the erysipelatous inflammations of the mucous membrane of the mouth, and it is for this disease that I particularly recommend it. In many of these cases in which I have used it, it has arrested the disease in its incipiency.

*Germantown, Tenn.*, Nov. 10th, 1848.

